# Current and Emerging Therapies for Uveitis in Axial Spondyloarthritis: A Scoping Review

**DOI:** 10.31138/mjr.310725.ect

**Published:** 2026-01-08

**Authors:** Dimitrios Deligeorgakis, Elpida Skouvaklidou, Vasileios Skepastianos, Evdokia Papadimitriou, Maria Boutel, Vasiliki Dimitriadou, Konstantinos Tsafis, Paraskevi Avgerou, Ioanna Katsigianni, Eleni Pagkopoulou, Christina Adamichou, Nikolaos Kougkas

**Affiliations:** 4th Department of Internal Medicine, Hippokration Hospital, Aristotle University of Thessaloniki, Thessaloniki, Greece

**Keywords:** axial spondyloarthritis, uveitis, monoclonal antibodies, review

## Abstract

**Aim:**

Uveitis is a common and potentially vision-threatening extra-musculoskeletal manifestation of axial spondyloarthritis (axSpA). Various biologic (bDMARD), as well as targeted synthetic (tsDMARD) disease-modifying anti-rheumatic drugs are established treatment options not only for axSpA, but for the whole spectrum of Spondyloarthritides. However, evidence from randomised-controlled trials (RCTs) specifically designed to assess axSpA-related uveitis treatment efficacy remains scarce, with the majority of evidence being extrapolated from RCTs with musculoskeletal orientation. This review examines current and emerging treatment options for axSpA-related uveitis, emphasising existing evidence gaps and clinical uncertainties.

**Methods:**

A scoping literature review was conducted following the PRISMA guidelines. PubMed, Scopus, and Cochrane databases were searched, according to the prespecified protocol criteria. After inaugural screening, eligible studies were evaluated performing quality assessment for final inclusion.

**Results:**

55 studies were retrieved and finally included. Studies were published between 2002–2025. Uveitis exposure-adjusted incidence rates per 100 patient years (EAIR/100PY) ranged between 0–4.5 for tumour necrosis factor inhibitors (TNFi), 0.5–3.9 for interleukin-17 inhibitors (IL-17i) and 0.8–3.3 for upadacitinib (UPA), as derived from available RCTs. Evidence from non-RCTs, reporting uveitis incidence rates per 100PY, ranged between 3.46–55.2 for etanercept, 1.38–15.7 for adalimumab, 1.82–25.9 for infliximab, 1.39–6.8 for golimumab, 0–9.4 for secukinumab and 0 for ixekizumab.

**Conclusion:**

Monoclonal TNFi are still the preferred therapeutic choice in axSpA patients with prior or high risk of uveitis. IL-17i are identified as second line treatment option especially in uveitis refractory to TNFi. Finally, JAKi remain a promising alternative, with more evidence needed to further establish their so far proven efficacy.

## INTRODUCTION

Axial spondyloarthritis (axSpA) is a chronic inflammatory rheumatic disease primarily affecting the axial skeleton, but it is also frequently associated with extra-musculoskeletal manifestations, such as uveitis, psoriasis, and inflammatory bowel disease (IBD). Among these, acute anterior uveitis (AAU) is the most common manifestation, occurring in approximately 25–40% of patients with axSpA during their lifetime.^1–3^ Uveitis not only contributes substantially to the disease burden but is quite often the initial clinical presentation, preceding it by years, particularly in individuals with HLA-B27 positivity.^4,5^

Epidemiological studies have consistently demonstrated that the risk of axSpA correlates strongly with HLA-B27 positivity, which is present in up to 90% of patients with ankylosing spondylitis (AS), the prototypical form of it.^6^ Uveitis in this population, typically manifests as unilateral, sudden-onset, recurrent anterior chamber inflammation, although intermediate involvement and more rarely panuveitis may also occur, sharing about the same clinical characteristics with psoriatic arthritis-related uveitis.^3,7^ Recurrent uveitis can lead to complications such as posterior synechiae, secondary glaucoma, macular oedema, and permanent visual impairment if not adequately managed.^8^ Other risk factors for uveitis that have been found, are positive family history, peripheral enthesitis, and IBD. Especially, IBD shows higher correlation rates with uveitis among other determinants.^9^ Despite its prevalence and morbidity, the therapeutic choices for uveitis in axSpA comparing to axial and peripheral involvement are restricted, leading to significant unmet needs in clinical management. Current joint recommendations by the Assessment of Ankylosing Spondylitis (ASAS) group and the European Alliance of Associations for Rheumatology (EULAR) for axSpA patients with previous or recurrent uveitis, favour monoclonal tumour-necrosis factor inhibitors (TNFi) over etanercept (ETN) and secukinumab (SEC).^10^ However, there is a lack of randomised controlled trials (RCTs) specifically designed to assess efficacy for ocular outcomes in axSpA, although the rates of AAU in the biologics treatment era are significantly lower.^9^

The absence of RCTs targeting axSpA-associated uveitis limits the development of clear, evidence-based treatment guidelines. Furthermore, head-to-head comparisons between biologic agents, including TNFi, Interleukin-17 inhibitors (IL-17i), and emerging small molecules such as Janus kinase inhibitors (JAKi), are lacking for ocular endpoints. These gaps in evidence are particularly concerning given the potential for irreversible vision loss if inflammation is inadequately controlled.^11,12^ Timely recognition and appropriate treatment of uveitis in axSpA are therefore essential to prevent cumulative ocular damage and preserve visual function. This requires not only therapeutic clarity but also close interdisciplinary collaboration between rheumatologists and ophthalmologists.^9^ The aim of this scoping review is to summarise the current literature on available and emerging therapies for uveitis in axSpA, highlight areas of therapeutic uncertainty, and discuss implications for clinical practice and future research.

## METHODS

### Literature search

The objectives, inclusion criteria, and methods for this scoping review were specified in advance and documented in a prespecified protocol (Supplementary material). This scoping review of the literature was conducted following the recommendations of the Preferred Reporting Items for Systematic Reviews and Meta-Analyses (PRISMA) extension for scoping reviews.^13^ The search was performed by two independent reviewers (ES and VS) in the following databases: PubMed, Scopus and Cochrane (first search: 08/04/2025). Search strings for the databases were structured by two reviewers (DD and ES) for each of the aforementioned databases, according to their syntax rules (Supplementary material). There were not any restrictions on publication date or language. Relevant alerts were created, and the articles published after the inaugural search, were also retrieved (up to 03/06/2025). ClinicalTrials.gov registry was also searched, as well as the reference lists of all retrieved articles.

### Inclusion and exclusion criteria

We included all trials (either randomised or not, along with their extensions), as well as observational studies reporting incidence rates per patient years (PY) of confirmed uveitis according to administered treatment, in patients diagnosed with axSpA. Studies included were restricted to adult patients. Only studies with biologic (bDMARDs) or targeted synthetic (tsDMARDs) disease-modifying anti-rheumatic drugs were included. Any patients with co-existence of other diseases able to provoke uveitis were excluded.

### Data extraction

At screening stage, titles and abstracts of all retrieved articles were reviewed independently by two reviewers (ES and VS), using the “Rayyan” systematic review production platform,^14^ and duplicates were removed. After duplicate removal, at screening stage, the reviewers independently reviewed the titles and abstracts of the articles according to the aforementioned inclusion and exclusion criteria. At the stage of eligibility, the two reviewers evaluated full texts, ending up to the eligible articles. Any discrepancies between the two reviewers were resolved with the assistance of the supervisor (NK).

Data extraction was conducted by one reviewer (DD). The following parameters were extracted: a) general publication details (first author’s surname, publication year), b) treatment administered, c) study design (RCT, open-label, cohort etc), d) setting (multicentre or single centre), e) diagnosis (AS, radiographic or non-radiographic axSpA), f) number of participants, g) Exposure-Adjusted Incidence Rates per 100 PY (EAIR/100 PY) and h) follow-up duration. When not given, EAIR/100 PY were calculated in cases of data availability. Extracted data were recorded on an Excel spreadsheet (Microsoft Corp., Redmond, WA, USA).

### Quality assessment

Quality assessment was conducted by two independent reviewers (KT and VD), using the relevant critical appraisal tools for RCTs,^15^ or the revised critical appraisal tool for the assessment of risk of bias for cohort studies,^16^ both proposed by the Joanna Briggs Institute, based on 13 or 11 relative questions respectively. Disagreements between reviewers were resolved through discussion and consensus.

## RESULTS

### Study Selection

**Figure 1.**
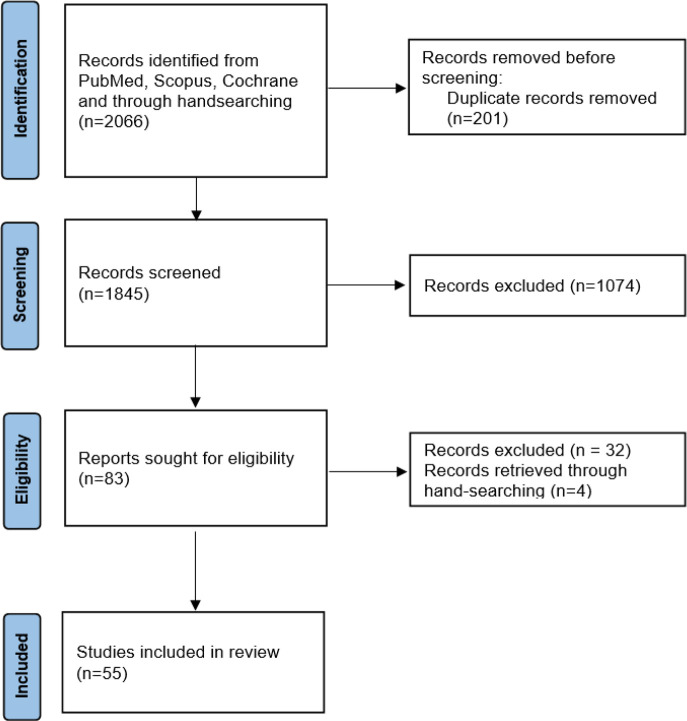
A flowchart presenting the literature search process, according to the Preferred Reporting Items for Systematic reviews and Meta-Analyses (PRISMA) statement. Source: Page MJ, et al. BMJ 2021;372:n71. doi: 10.1136/bmj.n71. This work is licensed under CC BY 4.0. To view a copy of this license, visit https://creativecommons.org/licenses/by/4.0/

Our initial search through databases yielded 2066 articles. After duplicate removal (201 duplicate were removed), 1762 articles were excluded at the stage of screening. 4 articles were retrieved through hand-searching. At the end, 55 articles were included in the review.^17–71^ A flowchart according to the PRISMA guidelines^72^ summarising the search procedure is presented in **Figure 1**.

### Characteristics of the included studies

The main characteristics of the studies finally included in the review are presented in **Table 1** and **Table 2**. All studies were published between 2002 and 2025. Most of the included studies were RCTs and had a multicentre setting. The studies’ duration ranged between 12 weeks to 5 years. Studies reported uveitis as EAIR/PY, incidence-rate (IR) or number of recorded cases.

**Table 1.** Randomised controlled trials of biologic and targeted DMARDs in axSpA, reporting uveitis events.

**Author**	**Year**	**Design**	**Setting**	**Population**	**Patients (n)**	**Uveitis measure (EAIR/100 PY)**	**Duration (weeks)**	**Conclusion regarding uveitis**
**Bimekizumab**								
Deodhar et al.^17^	2025	BE AGILE (RCT) & BE AGILE 2 (OLE)	MC	r-axSpA	303	0.7	256 (48 & 208)	Mild-moderate, no discontinuation
Baraliakos et al.^18^	2024	BE MOBILE 1 (RCT)	MC	nr-axSpA	244	1.5	52	3 cases (1 with uveitis history)
		BE MOBILE 2 (RCT)		r-axSpA	330	2.4		7 cases (5 with uveitis history)
Baraliakos et al.^19^	2025	BE MOVING (OLE)	MC	nr-axSpA & r-axSpA	518	1.3	52	6 cases (all with uveitis history)

**Upadacitinib**								
Van den Bosch et al.^20^	2025	SELECT AXIS-2 (RCT, OLE)		nr-axSpA	286	0.8	104 (52 & 52)	4 cases (1 with uveitis history)
								
Baraliakos et al.^71^	2025	SELECT AXIS-2 (RCT, OLE)	MC	bDMARD-IR AS	414	1	104 (14 & 90)	9 cases (3 with uveitis history, 5 serious, discontinuation of treatment in 2 during OLE)
Van der Heijde et al.^21^	2022	SELECT AXIS-1 (RCT, OLE)	MC	AS	182	3.3	104 (14 & 90)	16 uveitis events in 10 patients (9 with uveitis history, 1 discontinuation)

**Ixekizumab**								
Deodhar et al.^22^	2023	COAST-Y (RCT, OLE)	MC	nr-axSpA & r-axSpA	932	2.8	156 (52 & 104)	41 uveitis flares in 173 patients with uveitis history (IR 10.7/100 PY) 17 new onset of uveitis in 755 patients with no uveitis history (1/100 PY)
Deodhar et al.^23^	2020	COAST-X (RCT)	MC	nr-axSpA	198	1.9*	52	3 cases (all with uveitis history)
Dougados et al.^24^	2019	COAST-V (RCT)	MC	r-axSpA	641	3.9	52	20 cases (15 with uveitis history, 1 discontinuation)
		COAST-W (RCT)	MC					

**Certolizumab pegol**								
Van der Heijde et al.^25^	2021	C-axSpAnd (RCT)	MC	nr-axSpA	159	3.5	52	5 cases
		C-axSpAnd (OLE)			243	1.5	104	7 cases (5 with uveitis history)
Van der Heijde et al.^26^	2017	RAPID-axSpA (RCT, dose blind, OL)	MC	axSpA	315	4.5	204 (24 & 48 & 132)	Uveitis event rate at week 204 was 3.8/100 PY (n=218)

**Secukinumab**								
Poddubnyy et al.^27^	2021	SKIPPAIN (RCT)	MC	nr-axSpA & r-axSpA	380	0.7	24	2 cases
Baraliakos et al.^28^	2019	MEASURE 1 (RCT, OLE)	MC	AS	360	1.8	5 years (2 & 3)	24 cases (15 with uveitis history, 1 treatment discontinuation)
Marzo-Ortega et al.^29^	2020	MEASURE 2 (RCT)	MC	AS	211	0.5	5 years	4 cases of mild to moderate uveitis (3 with uveitis history)
Pavelka et al.^30^	2020	MEASURE 3 (RCT, OLE)	MC	AS	223	1.5*	3 years	8 cases (4 with uveitis history, 1 new onset) uveitis led to discontinuation)
Kivitz et al.^31^	2018	MEASURE 4 (RCT, OL)	MC	AS	346	1	104	6 cases (3 with uveitis history, no discontinuation)
Huang et al.^32^	2020	MEASURE 5 (RCT & OL)	MC	AS	456	2.2	52	10 cases, mild to moderate, no discontinuation
Deodhar et al.^33^	2020	PREVENT (RCT)	MC	nr-axSpA	543	1.2	104 (52 & 52)	9 cases (5 with uveitis history, mild to moderate, no discontinuation)
Baeten et al.^34^	2013	RCT	MC	AS	24	0	28	0 cases

**Golimumab**								
Van der Heijde et al.^35^	2022	GO-AHEAD (RCT, OLE)	MC	AS	189	0	60 (52 & 8)	0 cases
Deodhar et al.^36^	2018	GO-ALIVE (RCT)	MC	AS	105	0	28	0 cases
Deodhar et al.^37^	2015	GO-RAISE (RCT, OLE)	MC	AS	353	0	256 (24 & 232)	0 cases
Bao et al.^38^	2014	RCT	MC	AS	211	0.6*	56 (24 & 32)	1 case

**Adalimumab**								
Van der Heijde et al.^39^	2018	ABILITY-1 (RCT, OLE)	MC	nr-axSpA	190	0	156 (12 & 144)	
Landewé et al.^40^	2018	ABILITY-3 (RCT, OLE)	MC	nr-axSpA	673	3.3*	68 (28 & 40)	
Huang et al.^41^	2013	RCT, OL	MC	AS	342	0	24 (12 & 12)	0 cases
Van der Heijde et al.^42^	2008	ATLAS (RCT, OLE)	MC	AS	311	2.3*	104 (24 & 80)	12 cases (9 with uveitis history)

**Infliximab**								
Sieper et al.^43^	2014	INFAST (RCT)	MC	axSpA	105	0	28	0 cases
Inman et al.^44^	2010	RCT	MC	AS	39	0	52 (12 & 40)	0 cases
Barkham et al.^45^	2009	RCT	SC	AS	20	0	16	0 cases
Van der Heijde et al.^46^	2005	ASSERT (RCT)	MC	AS	202	0	24	0 cases
Marzo-Ortega et al.^47^	2005	RCT	SC	AS	28	6.8*	30	1 case
Braun et al.^48^	2002	RCT	MC	AS	34	13.3*	12	1 case (without uveitis history)

**Infliximab biosimilar**								
Park et al.^49^	2013	PLANETAS (RCT, OLE)	MC	AS	174	0.4*	102 (54 & 48)	1 case

**Infliximab / Etanercept**								
Giardina et al.^50^	2009	RCT	SC	AS	25	2*	104	1 case
					25	4*		2 cases

**Etanercept**								
Dougados et al.^51^	2017	EMBARK (RCT, OLE)	MC	nr-axSpA	111	1.6*	104 (12 & 92)	5 cases
Dougados et al.^52^	2014	SPARSE (RCT, OLE)	MC	axSpA	75	0	24 (8 & 16)	0 cases
Dougados et al.^70^	2012	SPINE (RCT, OLE)	MC	AS	77	0	24 (12 & 12)	0 cases
Barkham et al.^53^	2010	RCT	SC	AS	15	0	12	0 cases
Van der Heijde et al.^54^	2006	RCT	MC	AS	305	0	12	0 cases
Calin et al.^55^	2004	RCT	MC	AS	45	0	12	0 cases
Davis et al.^56^	2003	RCT	MC	AS	138	4.7*	24	3 cases
Brandt et al.^57^	2003	RCT, OLE	MC	AS	30	7.5*	30 (6 & 24)	1 case
Gorman et al.^58^	2002	RCT, OLE	MC	AS	20	0	10 months (4 & 6)	0 cases

* uveitis expressed as Exposure-Adjusted Event Rates (EAER) per 100 years, RCT: Randomised-controlled trial, OLE: Open-label extension, OL: Open-label, MC: Multicentre, SC: Single centre, AS: Ankylosing spondylitis, axSpA: Axial spondyloarthritis, r-axSpA: radiographic-axSpA, nr-axSpA: non-radiographic axSpA, EAIR: Exposure-Adjusted Incidence Rate, PY: Patient Years.

**Table 2.** Non-randomised controlled trials or cohorts of biologic DMARDs in axSpA, reporting uveitis events.

**Authors**	**Year**	**Treatment**	**Design**	**Setting**	**Population**	**Patients (n)**	**Uveitis measure**		**Duration**	**Conclusion regarding uveitis**
		ADA						1.38/100 PY (4.92%, 197/4004)	14256 PY	
		ETN						3.46/100 PY (11.7%, 245/2099)	7085 PY	for incident AAU prevention: adalimumab/golimumab > infliximab > secukinumab > etanercept
Kwon et al.^59^	2025	GOL	Retrospective nationwide cohort	MC	AS	34621	IR/100 PY (%, n (uveitis)/n (treatment receivers)	1.39/100 PY (4.0%, 85/2120)	6133 PY	
		IFX						1.82/100 PY (7.6%, 122/1603)	6710 PY	for recurrent AAU prevention:adalimumab > golimumab/infliximab/secukinumab > etanercept
		SEC						2.56/100 PY (4.6%, 15/325)	578 PY	
		IXE						0/100 PY (0%, 0/23)	15 PY	

Godzenko et al.^60^	2024	SEC	Retrospective	SC	AS	57	EAIR	9.4/100 PY	At least 12 months	Occurrences of uveitis were observed in 4 of 57 patients (7%) during SEC therapy and in1 of 22 (4%) patients during the NTK therapy.
		NTK				22	EAIR	4.8/100 PY		

Kwon et al.^61^	2024	ETN	Retrospective	SC	AS	99	Mean number of new onset uveitis incidents	17.9/100 PY	2 years	More frequent new-onset AU with ETN than ADA in AS patients
		ADA				68		4.7/100 PY		
		IFX				42		10.5/100 PY		

Gaffney et al.^62^	2023	SEC	SERENA, non-interventional ongoing	MC	r-axSpA	108	EAIR	0/100 PY	1.8 years (mean)	No new cases or flares of uveitis were reported during the study

Ramonda et al.^63^	2022	SEC	prospective, real-life cohort	MC	axSpA	249		1 case	24 months	1 discontinuation due to relapsing uveitis

Ahn et al.^64^	2022	ADA	Retrospective nationwide cohort	MC	AS	5938	IR/100 PY	4.8/100 PY	2 years	Increased HR for first AAU occurrence with ETN both for AAU +/- negative baseline history, compared to ADA, IFX and GOL
		ETN						8.4/100 PY		
		IFX						4.5/100 PY		
		GOL						4.6/100 PY		

Van der Horst-Bruinsma et al.^65^	2021	CZP	C - VIEW (OL)	MC	nr-axSpA & r-axSpA	89	IR/100 PY	17.7/100 PY	104 weeks	reduction of AAU flare event rate in patients with axSpA and a history of AAU from 97.5 to 17.7/100 PY
Lindström et al.^66^	2021	ADA	Retrospective nationwide cohort	MC	AS	4851	IR/100 PY (%, n (uveitis)/n (treatment receivers)	4/100 PY (11% 143/1249)	Therapy initiation between 1 Jan 2015 and 31 Dec 2018	Association of SEC with higher risk of AU compared to monoclonal TNFi, with similar risk compared to ETN
		ETN						7.5/100 PY (5% 104/1898)		
		IFX						2.9/100 PY (6% 54/883)		
		GOL						6.8/100 PY (10% 56/562)		
		CZP						4.5/100 PY (7% 25/335)		
		SEC						6.8/100 PY (6% 31/493)		

Van Bentum et al.^67^	2019	GOL	GO-EASY, prospective real-world	MC	AS	93		reduction of AAU event rate from 11.1 to 2.2/100 PY	12 months	Significant decrease in AAU incidence during GOL treatment in daily clinical practice

Lie et al.^68^	2017	ADA	Retrospective nationwide cohort	MC	AS	1365	IR/100 PY (%, n (uveitis)/n (treatment receivers)	15.7/100 PY (7.6% 31/406)	Therapy initiation between Jan2003 and Dec 2010	IFX and ADA present lower risk of AU than ETN
		ETN						55.2/100 PY (22.9% 81/354)		
		IFX						25.9/100 PY (13.1% 79/605)		

Rudwaleit et al.^69^	2009	ADA	RHAPSODY, prospective, OL, uncontrolled trial	MC	AS	1250	IR	7.4/100 PY	30 weeks	Reduction of AU flare rates from 15/100 PY before ADA to7.4/100 PY

ADA: Adalimumab, ETN: Etanercept, GOL: Golimumab, IFX: Infliximab, SEC: Secukinumab, IXE: Ixekizumab, NTK: Netakimab, CZP: Certolizumab-pegol, OL: Open-label, MC: Multicentre, SC: Single centre, AS: Ankylosing spondylitis, axSpA: Axial Spondyloarthritis, r-axSpA: radiographic axSpA, nr-axSpA: non-radiographic axSpA, IR: Incidence rate, PY: Patient Years, EAIR: Exposure-Adjusted Incidence Rate, AAU: Acute Anterior Uveitis, AU: Anterior Uveitis, TNFi: Tumour-necrosis factor inhibitor.

### Quality of the included studies

Although some aspects of certain non-RCTs were unclear, especially concerning the utilisation of strategies to address incomplete follow-up, the overall appraisal was satisfying for all studies included. The quality assessment of each study is presented in Supplementary material.

### Outcomes

Tumour-necrosis factor inhibitors [etanercept, infliximab (IFX), adalimumab (ADA), golimumab (GOL), certolizumab pegol (CZP)], IL-17i [secukinumab, ixekizumab (IXE), bimekizumab (BKZ)], and tsDMARD upadacitinib (UPA), where among the treatments analysed in the retrieved trials and cohorts. RCTs on TNFi were 27, on IL-17i 14 and 3 trials on UPA.

Concerning RCTs, the EAIRs/100 PY for TNFi, IL-17i and UPA ranged between 0–4.5, 0.5–3.9 and 0.8–3.3 respectively (**Table 1**). Among TNFi, RCTs of GOL reported almost 0 EAIR/100 PY, with only one case of uveitis in a total of 858 treated patients. IL-17i presented about the same uveitis rates between them, with the majority of uveitis episodes being experienced by patients with previous uveitis history. One trial of UPA (SELECT AXIS-2) reported 5 serious uveitis episodes, with 2 treatment discontinuations during the open-label extension (OLE) phase.

Four nationwide retrospective, 5 prospective and 2 smaller retrospective studies were identified among cohort studies. A large retrospective nationwide cohort with many PY on AS treatment reported uveitis incidence rates in a population of 34,621 South Korean patients (**Table 2**). ETN was associated with more uveitis incidents (3.46/100 PY) compared to other treatment options, whereas SEC was found to be less effective than TNFi in preventing both a new incident of AAU or recurrent AAU. Same results were reported by other 2 nationwide cohorts from Sweden, reporting higher risk of anterior uveitis in ETN treated AS patients (Table 2). Overall, IR / 100 PY ranged between 3.46–55.2 for ETN, 1.38–15.7 for ADA, 1.82–25.9 for IFX, 1.39–6.8 for GOL, 0–9.4 for SEC and 0 for IXE (15 PY of analysis, **Table 2**).

## DISCUSSION

### TNFi

TNFi therapies remain the cornerstone of biologic treatment for axSpA, and their role in managing and preventing AAU is of particular clinical relevance. Data from both RCTs and real-world observational studies suggest important differences among TNFi in their effectiveness for uveitis prevention.

Among monoclonal antibody TNFi agents, ADA and IFX consistenly show superior efficacy in reducing the incidence and recurrence of uveitis. In the ATLAS trial, ADA was associated with a uveitis incidence rate of 2.3/100 PY, with most events occurring in patients with a prior history of uveitis.^73^ Observational studies support these findings; in a large Korean cohort study involving over 34,000 patients with AS, ADA had the lowest incidence rate (1.38/100 PY) compared to IFX (1.82/100 PY), GOL (1.39/100 PY), and ETN (3.46/100 PY).^59^ For recurrent AAU, ADA was again superior, followed by GOL, IFX, and SEC, with ETN showing the least benefit.^59^

ETN appears to be less effective for preventing uveitis. In multiple RCTs including EMBARK,^51^ SPARSE, ^52^ and others,^53–58,70^ uveitis events were rare or absent — likely due to patient selection and study duration. However, real-world data provide a clearer picture: ETN was associated with the highest incidence rates of both new-onset and recurrent uveitis, reaching up to 55.2/100 PY in historical cohorts,^66,68^ and 17.9/100 PY in more recent ones.^59^ This large variability could be possibly explained due to population variations or different treatment approaches. However, these findings highlight ETN’s limited efficacy for ocular inflammation, despite effective axial symptom control.

GOL has demonstrated an intermediate profile. While RCTs such as GO-AHEAD,^35^ GO-ALIVE^36^ and GO-RAISE,^37^ reported zero or very low incidence of uveitis events, observational studies indicate it as more effective than ETN, but less than ADA in preventing AAU. A prospective real-world cohort (GO-EASY) showed reduction in uveitis flares from 11.1 to 2.2/100 PY during GOL treatment,^67^ confirming its potential in clinical practice.

IFX also demonstrated strong efficacy, with early trials like INFAST^43^ and ASSERT^46^ reporting no uveitis events during study periods. However, more relevant data come from observational cohorts; IFX consistently showed lower uveitis rates than ETN, with IRs ranging from 2.9 to 25.9/100 PY depending on population and time period.^59,61,66,68^ These results affirm IFX’s suitability in patients with uveitis-prone axSpA. CZP, though newer in the axSpA landscape, has shown compelling results in uveitis prevention. The C-VIEW study reported a dramatic decrease in AAU flare rates from 97.5 to 17.7/100 PY over 2 years of CZP therapy in patients with a history of uveitis.^65^

### IL-17i

IL-17i of interest for this scoping review comprise of SEC and IXE, selective monoclonal antibodies against IL-17A and BKZ, a dual inhibitor of IL-17A and IL-17F. These IL-17i are approved for treating radiographic ax-SpA (r-axSpA) and non-radiographic axSpA (nr-axSpA) as a first line therapy or after inadequate response/intolerance to other bDMARDs or tsDMARDs.^10^

### Secukinumab

SEC was the first IL-17i licensed for ax-SpA and thus more widely studied for its impact in uveitis compared with IXE and BKZ. Evidence of efficacy in uveitis is drawn from primary double-blind placebo controlled RCTs and their corresponding OLE, powered and prioritised to investigate axSpA disease activity. However, interesting insights are presented from retrospective and prospective cohorts including investigations about eye inflammation on their registries.^27–34,59,60,62,63,74–77^

In PREVENT RCT,^33^ 555 patients with nr-axSpA received SEC or placebo of whom 12.7% of active group and 9.7% of placebo group had a history of uveitis. At the end of blind session (first 20 weeks) only 0.5% of participants had another episode of uveitis and SEC superior to placebo at 108 weeks with EAIRs of 1.2/100 PY (9 patients) versus 1.8/100 PY (2 patients).^33^ During MEASURE 1–3 trials 794 AS patients were exposed to SEC therapy with 17% and 74% of them having at baseline a previous episode of uveitis and HLA-B27 positive gene respectively. The EAIR of uveitis in MEASURE 1 (5 years) was 1.8/100 PY, in MEASURE 2 0.5/100 PY and in the pooled MEASURE 1–3 trials EAIR was 1.4/100 PY.^28–30,34,74–77^ Another two MEASURE trials were conducted, MEASURE 4 and MEASURE 5, in which the active arm had similar rate of uveitis episodes with placebo during the placebo-controlled time (16 weeks), with EAIRs ranging from 1 to 2.2/100 PY in SEC groups for the entire study period.^31,32^ Similar results are presented from SKIP-PAIN trial employing 380 (81% HLA-B27 positive, 15% comorbid uveitis) r-axSpA and nr-axSpA patients to receive SEC or placebo for 8 weeks and then another 16 weeks only SEC in a blinded fashion, with no incidence of uveitis during the placebo-controlled time and an EAIR of 0.7/100 PY for the entire period (24 weeks).^27^

Pooled results from the placebo-controlled time of MEASURE 1–5 and PREVENT RCTs highlight a low EAIR of 1.29/100 PY in the SEC 150 mg group whereas 1.72/100 PY in the placebo group. No uveitis events were observed in SEC 300mg group. Overall, HLA-B27 rate and previous uveitis history were comparable for SEC and placebo (75.9% vs 75.7% and 13% vs 15% respectively).^78^ While evidence from RCTs suggests a low incidence of uveitis in SEC-treated AS patients, data from large-scale cohorts are controversial and SEC documented performance is inconsistent.^59,60,62,63,66^

A large prospective Italian cohort of 246 axSpA patients (40.9% HLA-B27 positive) with 14 (5.6%) experiencing a previous uveitis event, reports good overall tolerability of SEC. The Italian cohort followed this more diverse than RCTs axSpA population for a total of 24 months and identified a single patient who discontinued treatment due to recurrent episodes of uveitis; however, the total number of uveitis incidences is not reported.^63^

Furthermore, SERENA, an international study from UK, reports EAIRs of 0/100 PY after a two-year follow-up of 108 r-axSpA patients (4 of them having baseline co-morbid uveitis).^62^

On the other hand, when 73 patients with AS [19 (26%) had comorbid uveitis], treated with SEC or netakimab were followed for at least a year, the incidence of uveitis for those who had previously had uveitis seemed to rise when exposed to IL-17i. Importantly, EAIRs for patients receiving SEC was 10.1/100 PY before the start of any bDMARD therapy and 9.4/100 PY during SEC therapy and for patients with baseline uveitis was 22.5/100 PY before treated with bDMARDs and 29.1/100 PY during IL-17i treatment (SEC and netakimab), although this was not a significant difference.^60^

Insightful results are also found from two retrospective nationwide multicentre cohorts of patients. The larger one involved 34,621 AS patients without uveitis history, treated with bDMARDs (ADA, ETN, GOL, IFX, SEC). During treatment with SEC, hazard ratio (HR) for uveitis incidence was 1.32, compared with a bDMARDs not exposure group and 2.17 compared with ADA exposure group. For recurrent uveitis, HR was 1.26 compared with a bDMARDs not exposure group. These results led the authors to propose a possible drug hierarchy which places SEC after monoclonal TNFi for incident uveitis prevention although total exposure time for IL-17i was short.^59^

The other cohort consisted of 4,851 AS patients without uveitis for at least one year prior to registration, who were also undertaking treatment with bDMARDs. Higher SEC HR of uveitis were reported (EAIR 6.8/100 PY, similar to ETN) versus monoclonal TNFi and estimates a HR of SEC versus ADA equal to the latter cohort for first uveitis incidence (2.32).^66^

Devoid of RCTs powered to investigate treatment performance in active uveitis and based on the available evidence, SEC is inferior to monoclonal TNFi when co-morbid uveitis is concerned.

### Ixekizumab

Data for IXE impact on uveitis are presented in COAST V, W, X and Y trials, the original IXE double-blind RCTs for axSpA. OLE studies also provide additional information.^22–24,79^ These results are not so optimistic for IXE. In COAST V (bDMARD naïve axSpA patients) and COAST W (TNFi experienced) a total of 641 patients received IXE for approximately 52 weeks. Safety analysis for uveitis calculated an EAIR of 3.9/100 PY, inferior to SEC, although baseline uveitis history in COAST V and W (21–23%) and HLA-B27 positivity (82–91%) were slightly higher than in the SEC RCTs.^24^ In COAST X placebo was retained up to 52 weeks in 303 (105 placebo, 198 IXE) nr-axSpA patients. Comorbid uveitis history was found in 11% of each group, however IXE performed equally to placebo in terms of uveitis episodes.^22^ When COAST V, W, X are considered, an EAIR of 3.3/100 PY was estimated, generated by 52 patients, 5.3% of all the participants, who experienced uveitis during trials.^79^ Finally, COAST-Y followed patients originating from COAST V, W, X for an additional year (cumulative time in study up to 3 years). Safety outcomes reported an EAIR of 2.8/100 PY for uveitis, mostly mild and moderate flares but these EAIR raised to 10.7/100 PY for patients with comorbid uveitis history whereas dropped to 1.0/100 PY for those with no uveitis history. IXE may be regarded as inferior to SEC in terms of diminishing uveitis occurrence in axSpA patients, although caution is needed when interpreting the data, especially for HLA-B27 gene and baseline uveitis cases.^23^

### Bimekizumab

The novel IL-17 antagonist and the only one simultaneously blocking IL-17 A and F is bimekizumab. BKZ was first approved for the treatment of plaque psoriasis and later for axSpA, based on significant improved axSpA activity indices from BE-MOBILE, BE MOVING and BE-AGILE randomised controlled RCTs and OLE. These trials also offer us limited but valuable information about uveitis activity and relapses.^17–19^ In BE-MOBILE 1 (254 nr-axSpA patients, 16% prior uveitis) and 2 (332 r-axSpA patients, 17% prior uveitis) undertook BKZ. During the placebo-controlled time (through 16 weeks), BKZ performed better than placebo and uveitis was reported in 2 vs 6 patients in BE-MOBILE 1 and in zero vs 5 patients in BE-MOBILE 2, calculating an EAIR of 1.8/100 PY for BKZ vs 15.4/100 PY for placebo.^80^ After re-randomising placebo patients into active groups (weeks 16 to 52), EAIRs measured for uveitis were 1.5/100 PY for nr-axSpA, 2.4/100 PY for r-axSpA and 2.6/100 PY for all axSpA patients for the whole study period. When stratified by their prior uveitis history nr-axSpA patients with a positive history conferred an EAIR of 3.2/100 PY and those with a negative history an EAIR of 1.2/100 PY while for nr-axSpA patients EAIRs were 10.8/100 PY and 0.8/100 PY respectively. After another 52 weeks of follow-up, BE-MOVING trial reports an EAIR for the whole study cohort of 1.3/100 PY (weeks 52–104).^19^ BE-AGILE had a similar design and included 303 r-axSpA patients with high HLA-B27 positivity (89%) and while 15% had a previous history of uveitis. Follow-up was long (256 weeks) and resulted in an EAIR 0.7/100 PY for uveitis.^17^ Combining results from BE-MOBILE, BE-MOVING and BE-AGILE (RCTs and OLE, N=848) present an EAIR of 1.2/100 PY.^80^ Results so far imply that BKZ might be non-inferior to SEC as they present comparable EAIRs across various studies, however, it has yet to show real-life evidence of efficacy through large cohort studies. BKZ seems to confer a protective effect for patients who never had uveitis.

### JAKi

UPA and tofacitinib (TOFA) are included in axSpA therapeutic armamentarium and while both are licensed for treating r-axSpA, only UPA is indicated for nr-axSpA. Evidence for efficacy in eye inflammation is limited for UPA, extrapolated from drug original RCTs (SELECT AXIS)^20,21,71,81–83^ and scarce for adult SpA population receiving TOFA (case reports).^84^ SELECT axis 1 is a multi-centre RCT which investigated UPA for biological naïve r-axSpA patients, primarily exploring the now well-known positive arthritic response and safety concerns. 178 patients entered the OLE study and over the course of 2 years 10 patients exhibited recurrent, mild to moderate and topical-treated uveitis episodes (9 of them had a history of uveitis) with a total of 16 incidents. The EAIR for patients with, without history of uveitis and for the whole group were 3/100 PY, 0.3/100 PY and 3.3/100 PY respectively, with a cumulative exposure adjusted events ratio (EAER) of 5.2/100 PY. HLA-B27 positivity was 75–78% in this patient group, however baseline medical history of uveitis is not recorded in the original RCT imposing difficulties to draw more meaningful conclusions of the impact of UPA in patients who did not experience a flare.^21,81^ Van der Heije et al. in SELECT AXIS 2 explored the effect of UPA in 414 bDMARDs-inadequate responders r-axSpA patients with baseline uveitis in 15 (7%) and 21 (10%) patients of placebo and UPA group. HLA-B27 was positive in 81–85% of patients. Upadacitinib had almost three-fold better efficacy in reducing flares compared to placebo (0.5% vs 1.4% of patients flared in UPA and placebo group respectively) during the double-blind placebo-controlled trial time (14 weeks). In the OLE (2 years duration) nine uveitis attacks occurred overall (EAER 1.3/100 PY), three patients had relapsing attacks (1 in original UPA and 2 in original placebo groups), five of them were regarded serious, leading to discontinuation of treatment in two patients.^81,82^

Similarly, in SELECT axis 2 involving 286 patients with nr-axSpA, 11 (7%) and 12 (8%) of placebo and UPA group had a previous uveitis episode. UPA also led to fewer flares, with 1.3% of the active group having a uveitis relapse versus 1.9% in the placebo group (52 weeks duration). After 2 years in OLE study, patients presented with EAER of 1.1/100 PY, attributed to four uveitis attacks (one in a patient with prior history).^83^

Notwithstanding the lack of evidence from real-life large-scale cohort or prospective studies so far, there are some implications from RCTs, designed to measure arthritic disease activity for a positive effect of UPA in reducing uveitis relapses, especially when compared with EAIR from other bDMARDs (ETN, IXE) and in relation to placebo. Knowledge about the status of uveitis (active or in remission) at the time of UPA initiation would be beneficial but is not addressed due to the different methodology and primary goals of these UPA studies.

Finally, a phase 4 open-label multicentre study (UPFOR-U, NCT07018206, not yet recruiting) is going to specifically evaluate the impact of UPA on the frequency of AAU in adults with axSpA, with a prior AAU history in the past 52 weeks.

This review is subject to several limitations. Firstly, there is a notable scarcity of RCTs specifically focusing on axSpA-related uveitis. Aside from the aforementioned registered trial that is yet to commence recruitment, no additional future studies appear to be planned. Additionally, earlier studies, particularly those conducted prior to 2010, had a rather short follow-up period, limiting the ability to assess long-term outcomes. Furthermore, significant heterogeneity exists between the included studies, mainly in terms of terminology (AS, r- or nr axSpA), the concomitant cDMARDs, topical drug administration and rescue therapies, all of which complicate direct comparisons. The considerable diversity in study designs, along with variations in patient population (e.g. prior history of uveitis, disease duration, other extra-musculoskeletal manifestations), raise substantial concerns regarding heterogeneity of the included studies, thereby limiting the feasibility of conducting a systematic review with meta-analysis.

## CONCLUSION

This review raises the awareness to clinicians about the appropriate choice of the available treatments for axSpA complicated with uveitis. Taken together, these findings suggest a class effect favouring monoclonal TNFi agents (ADA, IFX, GOL, CZP) over ETN for uveitis management. The consistent underperformance of ETN — especially in real-world data — underscores the importance of phenotype-driven treatment selection in axSpA. In patients with prior or high risk of AAU, monoclonal TNFi should be prioritised. IL-17i were identified as second line treatment options with great results in relapsing TNFi experienced uveitis, with evidence suggesting a protective effect against new uveitis episodes with dual IL-17 A/F inhibition. All in all, JAKi (mainly UPA) are a quite promising drug class for the treatment of uveitis as a third line therapy, necessitating though more trials to reinforce this writers’ statement. Further head-to-head comparisons and long-term real-world studies are needed to validate these trends and refine clinical decision-making in axSpA-associated uveitis.
